# Marine Collagen Substrates for 2D and 3D Ovarian Cancer Cell Systems

**DOI:** 10.3389/fbioe.2019.00343

**Published:** 2019-12-13

**Authors:** Francesca Paradiso, Joan Fitzgerald, Seydou Yao, Frank Barry, Francesca Taraballi, Deyarina Gonzalez, R. Steven Conlan, Lewis Francis

**Affiliations:** ^1^Reproductive Biology and Gynaecological Oncology Group, Swansea University Medical School, Swansea, United Kingdom; ^2^Center for Biomimetic Medicine, Houston Methodist Research Institute, Houston, TX, United States; ^3^Regenerative Medicine Institute (REMEDI), National University of Ireland Galway (NUI), Galway, Ireland

**Keywords:** collagen, jellyfish, biocompatibility, ovarian cancer, cell culture

## Abstract

A fundamental structural component of extracellular matrix in all connective and interstitial tissue, collagen is the most abundant protein in the human body. To date, mammalian collagens sources represent the golden standard for multiple biomedical applications, while marine-derived collagens have largely been used in industry (food, pharmaceutical, and cosmetic), with little use in research and clinical applications. Herein we demonstrate the effective use *Rhizostoma pulmo* jellyfish collagen, a source of biocompatible, sustainable collagen for 2D and 3D cell culture, addressing the global drive for technological developments that result in the replacement of animals and their derived products in research. Jellyfish collagen harbors similar structural features mammalian collagen type I, despite differing slightly in amino acid content. Jellyfish collagen supports ovarian cancer (OvCa) cell line proliferation, cellular morphology and expression of epithelial to mesenchymal transition markers, supporting the use of *R. pulmo* as a non-mammalian collagen cell culture substrate. Furthermore, *R. pulmo* collagen is effective in 3D device fabrication such as sponges where it mimics tissue architecture complexity. OvCa cells migrated and differentiated within the *R. pulmo* collagen 3D scaffolds confirming its suitability for advanced cell culturing applications, providing an excellent alternative to mammalian collagen sources for the culture of human cells.

## Introduction

Collagens represent 30% of total protein mass in mammals, providing a fundamental structural component of extracellular matrix (ECM) in all connective and interstitial tissue (Gelse et al., 2003). Since the discovery of collagen II by Miller and Matukas (1969), 26 new collagen types have been discovered, which have been classified into subfamilies based on their supramolecular assembly, namely fibril-forming collagens, fibril-associated collagens, network-forming collagens, anchoring fibrils, transmembrane collagens, basement membrane collagens and others with unique functions (Gelse et al., 2003).

Fibril forming collagens share a common structural feature, the triple helix, which can make up 96% of their structure (collagen I) to <10% (collage XII) (Ricard-Blum, 2011). The triple helix is composed of three polypeptide α chains, composed of the peptide triplet repeat Gly-X-Y (X = proline, Y = hydroxyproline), conserved structural features which are crucial in mediating the spectrum of collagen functions (Gelse et al., 2003). Biological sources of collagen type I include mammals skin, porcine/bovine/ovine tendon tissue and rat tail, while bovine, porcine and chicken cartilage tissues provide a good source for collagen type II. The ability of these collagen sources to be fabricated into varying scaffold forms such as hydrogels, sponges, fibers, films, and hollow spheres provide tools for mimicking complex biological and mechanical features of native tissue (Sorushanova et al., 2019). Furthermore, by tuning scaffold porosity, shape and topography, clinicians can have access to a powerful array of controlled structures for tissue grafting, that can promote cell growth/differentiation. Additionally, bioinspired collagen-based *in vitro* culture methods provide a base for ECM substitutes in pathologic models for drug screening (Sorushanova et al., 2019).

Mammalian collagen devices are used in many biomedical applications due to their excellent biocompatibility, high biodegradability and good mechanical, haemostatic, and cell-binding properties (Lee et al., 2001). Conversely, complex collagen extraction methods, together with limited and expensive collagen sources, and the risk of infection with transmissible diseases such as spongiform encephalopathy, transmissible spongiform encephalopathy, and foot and mouth disease, have led to the exploration of alternative functional collagen sources with low immunogenicity and reduced risk of causing transmissible disease (Felician et al., 2018).

Many non-mammalian species, both vertebrate and invertebrate, have been evaluated as new and alternative collagen sources. Such collagen sources have predominantly been used in different applications including bone tissue engineering and related diseases, and cosmetic and/or skin care (Silva et al., 2014; Rahman, 2019). To date, marine-derived collagens have largely been used in the food, pharmaceutical and cosmetics industries, and to a much lesser degree for biomedical research and clinical applications (Parenteau-Bareil et al., 2010). Marine species include invertebrates such as cuttlefish, sea anemone, prawn, star fish, sponges, sea urchin, octopus, squid or vertebrate like fish, and marine mammals have been evaluated (Felician et al., 2018). Extraction of collagen from jellyfish species has been limited to *Somolophus meleagris* (Nagai et al., 1999; Song et al., 2006), *Rhizostomous* jellyfish, *Chrysaora* sp. jellyfish (Barzideh et al., 2014), and *Rhopilema esculentum* (Hoyer et al., 2014). Of these, collagen derived from *Rhizostoma pulmo* has been shown to have a high degree of similarity to mammalian type I collagen (Addad et al., 2011).

Fibril forming collagen type I is the major component of tissue ECM, exerting both mechanical and biological functions. It contributes to tissue architecture and strength while interacting with cells through several receptors, promoting cells growth, differentiation and migration (Ricard-Blum, 2011). In a tumor setting collagen remodeling (degradation and redeposition) strongly affects tumor infiltration, angiogenesis, invasion and migration (Provenzano et al., 2006; Fang et al., 2014).

Epithelial ovarian cancer is the fifth leading cause of cancer-related mortality in women and the most lethal gynecological malignancy (Cho et al., 2015). Dysregulation in collagen deposition or its degradation is implicated in ovarian cancer (OvCa) progression. Normal ovarian tissue has a specific collagen signature characterized by thin, long wavy fibrils, parallel to the epithelial boundary, while, during cancer progression collagen organization facilitates cancer cell migration by creating a net of thick and short fibrils, usually perpendicular to the epithelial/cancer growing boundary, known as Tumor-Associated Collagen Signature (TACS-3) (Adur et al., 2014; Cho et al., 2015). Collagen remodeling and physical reorganization not only exert a pro-migratory function but are also associated with chemoresistance (Gurler et al., 2015).

Here we have undertaken a comprehensive evaluation of *R. pulmo* collagen to determine its utility in supporting OvCa cell growth, proliferation and migration. We characterized *R. Pulmo* collagen structure and aminoacidic composition, we then used it as coating or scaffold to understand its suitability as support for both 2D and 3D cell culture systems (Barbolina et al., 2007; Mitra et al., 2011; Mckenzie et al., 2017). As a sustainable alternative to mammalian/vertebrate sources, which serves to deliver advances in the communities' desire to reduce its reliance and impact on the use of mammalian species and their derived products, *R. pulmo* derived collagen offers a reliable substrate for *in vitro* studies, physiologically recapitulating cancer cell environments.

## Materials and Methods

### Collagen Sources

Type I collagen from *Rhizostoma pulmo* jellyfish (© 2019 Jellagen), type I collagen from rat tail (Millipore) and type I collagen from bovine (Sigma-Aldrich) were used as references.

### Cell Culture

SKOV.3 (ATCC, Virginia, USA) cells were grown in McCoy's media supplemented with 10% FBS (10500-064, Gibco) and 1% Pen Strep (15140-122, Gibco); OVCAR.3 (ATCC, Virginia, USA) were grown in RPMI media supplemented with 20% FBS (10500-064, Gibco), 1% Pen Strep (15140-122, Gibco), insulin 0.01 mg/ml.

### SDS Page

SDS-PAGE was performed according to the method of using a 4–20% gradient. Samples were mixed with Laemmli sample buffer (Bio-Rad) with b-mercaptoethanol and heated for 5 min at 95°C. Different volumes of *Rhizostoma pulmo* collagen solution and 30 ug of rattail (rt) and bovine (bv) collagen were loaded to the gel and run at 100 V for 10 min followed by 120 V for 1.5 h. Following electrophoresis, protein bands were stained with Coomassie brilliant blue R- 250. Prestained-dual color marker (Biorad) was used to estimate the approximate molecular weight of collagen samples. Type I collagen from rat tail (Millipore) and type I collagen from bovine (Sigma-Aldrich) were used as references.

### Western Blotting

When confluent, cells were scraped into cold lysis buffer (RIPA buffer from Thermo Fisher Scientific) and a mixture of protease inhibitors (P8340, Sigma) while on ice. Cellular lysates were clarified, and protein was quantified by DC™ (detergent compatible) protein assay (Bio-Rad, Richmond, CA). Proteins (10 μg) were separated on a 10% SDS-polyacrylamide gel and blotted onto a PVDF membrane. PVDF membrane were blocked for 1 h at room temperature with 5% BSA in Tris-saline buffer containing 0.02% Tween-20 and incubated in primary antibody (1:1000 of E-cadherin ab1416; N-cadherin ab12221; Vimentin sc-6260; GAPDH sc-47724) overnight at 4°C. After washing in TBST, blots were incubated for 1 hr at room temperature with mouse or rabbit IgG HRP (1:2000 of NA931V or NA934V, Ge Healthcare) and the immunoreactive complexes visualized by the ECL Western blotting system, using the ChemiDoc™ Imaging System.

### Amino Acid Sequencing

We analyzed acid solubilized collagen derived from *Rhizostoma pulmo* tentacles (3.8 mg/ml in 0.1 M acetic acid), Type I collagen from rat tail (4.19 mg/ml, Millipore) and type I collagen from bovine (Sigma-Aldrich). One milligram of each sample was placed in 1.5 ml microcentrifuge tubes, freeze dried overnight, resuspended in 200 μl of 6N constant boiling HCl (Thermo Scientific) and transferred to a vacuum hydrolysis tube (Thermo Scientific). The hydrolysis tube was purged with nitrogen, evacuated and sealed. Samples were heated for 22 h at 110°C in an oven to enable hydrolysis, the vacuum was released and the HCl was evaporated by placing the open tube in an oven at 60°C for 30–40 min. The hydrosylate was resuspended in 150 μl of lithium loading buffer (Biochrom) and transferred to a 1.5 ml microcentrifuge tube. Hydrolyzed samples were transferred to glass vials and loaded in the autosampler tray after a dilution of 1:5. An injection volume of 20 ul hydrolyzed protein was analyzed for each sample. In addition, 40 μl of amino acid standard (A9906, Sigma) and 40 μl of loading buffer (a blank solution) were analyzed. Absorbance was read at 570 and 440 nm.

### Fourier Transform Infrared (ATR-FTIR) Spectroscopy

Fourier transform infrared spectra of freeze-dried *R. pulmo* collagen were obtained using a Perkin Elmer FTIR spectrometer. Infrared spectra were recorded in the range of 4,000–400 cm^−1^ at an aperture of 1 and sensitivity of 1.5.

### 2D Coating Plates Preparation

Corning 6 well plates and Nunc™ Lab-Tek™ 8-well Chambered Coverglass (ThermoScientific) were coated with rat tail collagen and *R. pulmo* collagen. Ninety microgram of collagen was used to coat 6 well plates for In Cell analysis, WB, RT-PCR and 7.6 ug for 8-well Chambered Coverglass to perform immunofluorescence staining. Collagen specific amount was added on each plate and leaved overnight at 4 degree. Next day, supernatant was collected and the plate left at 4 degree until use (they are stable for 1–2 days). Before seeding the cells, plate were washed with PBS and after dried under the hood for 20 min. Finally the plate was sterilized turning turn the UV on.

### In Cell Analysis

InCell Analyser 2000 (GE Healthcare) was used to analyze number of cells on different coated plates. Following culture period media was removed from monolayer cultures and washed with PBS. Cells were seeded on pre-coated plates and grew up to 5 days before analysis. Cells grown for 1–2–3–4–5 days were stained with Hoescht 33342 (Life Technologies Corporation) (dilution of 1:2000 from a 10 mg/ml solution in water) in normal media and incubated at room temperature for 10 min to stain the nuclei. Cells were immersed in PBS for analysis in the InCell Analyser 2000. Random distribution of fields across the surface of the well was used to capture 30 fields/well. Images were analyzed using InCell Developer (GE Healthcare) to quantify number of cells using DAPI staining.

### RNA Extraction From Collagen Scaffolds

To collect scaffold, we washed it with PBS, and freeze quickly (1 min) in a hexane bath immersed on dry ice. We stored them at −80 degree. To fully disrupt the scaffold, we submerged it in lysis buffer (RLT, RNeasy Mini Kit, Qiagen) and we used TissueRuptor II (Qiagen) for 20 s maximum at full speed.

### DNA Extraction From Collagen Scaffolds

We used Papain from papaya latex (P3125, Sigma) to digest *R. pulmo* scaffolds. A buffer made up of 300 ug/ml of papain, 2 mM DTT, 20 mM NaAc ph 6.8, 1 mM EDTA was used to incubate the sample at 60 degree for up to 2 h. Quant-iT picogreen dsDNA kit (Invitrogen) was used to assess double-stranded DNA in solution. Samples were read at the emission of 520 nm.

### RT-PCR

RNA was extracted from cells grown on 2D coated plates or 3D collagen scaffolds using RNeasy Mini Kit (Qiagen) according to the manufacturer's instructions. Hundred nanaogram (cells grown on 3D collagen scaffolds) or 1 ug (cells grown on 2D coated-plates) of total RNA were reverse transcribed into cDNA using the kit from Applied Biosystem. Primer sequences for each gene are summarized below. GAPDH and RLP19 were used as internal references for normalization. Quantitative polymerase chain reaction (qPCR) was undertaken using CFX96 Real Time PCR Detection system (Bio-Rad, UK) and analyzed using relative AACt method.

**Table:** 

MT1-MMP	FW: 5^′^-GAGACACCCACTTTGACTC-3^′^
	REV: 5^′^-CAGCCACCAGGAAGATGTC-3^′^ s
COL11A1	FW: 5^′^-ACCTGACCTGCCGTCTAGAA-3^′^
	REV: 5^′^-TCCACCACCCTGTTGCTGTA-3^′^
Snail	FW: 5^′^-ATCGGAAGCCTAACTACAGCGAGC-3^′^
	REV: 5^′^-CAGAGTCCCAGATGAGCATTGG-3^′^
E cadherin	FW: 5^′^-TTATGATTCTCTGCTCGT-3^′^
	REV: 5^′^-TCTTTGTCTGACTCTGAG-3^′^
Vimentin	FW: 5^′^-GAGAACTTTGCCGTTGAAGC-3^′^
	REV: 5^′^- TCCAGCAGCTTCCTGTAGGT-3^′^
Yap	FW: 5^′^-TGAACAAACGTCCAGCAAGATAC-3^′^
	REV: 5^′^-CAGCCCCCAAAATGAACAGTAG-3^′^

### Scaffold Molding and Cell Seeding

Eight hundred microgram of collagen/well of a Costar 96 plate (flat bottom) was used to mold scaffolds. Collagen samples were lyophilized after collagen deposition on the plates. Samples were frozen at −20°C and then lyophilization was achieved using a Scanvac Coolsafe 55-9 freeze drier (Labogene, Denmark). After, scaffold were crosslinked using 1-ethyl-(3-3-dimethylaminopropyl) carbodiimide hydrochloride (EDC) (cat no: E1769, Sigma Aldrich) in 80% ethanol at 1% w/v for 90 min. Cross-linked scaffolds were rinsed in deionized water three times and left in 1% glycine overnight, at room temperature, to quench the reaction. Finally, constructs were lyophilized again to get all the residual liquid out and preserve their cylindrical shape.

### Scanning Electron Microscopy (SEM) and Pore Size Analysis

Freeze-dried collagen scaffolds were examined using scanning electron microscopy (SEM) (JSM, Jeol, Japan). Scaffold pore size was detected using SEM software (Hitachi). A total of 10 pores were analyzed per images and a total of 3 images were analyzed within 3 different scaffolds.

### Haematoxylin and Eosin Staining

Collagen scaffolds seeded with cells were fixed in formalin 4% for 24 h at 4 degree and then washed 2 times in PBS and embedded in paraffin. Microtome was used to cut 5 um sections form each sample. Haematoxylin and Eosin (TSC biosciences, UK) staining was performed following these steps: 10′ in Histochoise (Sigma), 4′ EtOH 100%, 4′ EtOH 95%, 4′ EtOH 70%, 4′ dH20, Haematoxylin staining (tcs biosciences, 1:2 dilution in dH20) 1′ and wash with water. Eosin staining (tcs biosciences, 1:5 dilution in Dh20) 2′, wash with water, 2′ EtOH 70%, 2′ EtOH 95%, 2′ EtOH 100%, 5′ Histochoise (Sigma) and mounted with DPX.

### Picrosirious Red Staining

Collagen scaffolds seeded with cells were fixed in formalin 4% for 24 h at 4 degree and then washed 2 times in PBS and embedded in paraffin. Microtome was used to cut 5 um sections form each sample. Collagen fibrils were indicated histologically with picrosirius red staining. Sections were hydrated through descending concentrations of ethanol and stained with 0.1% (w/v) picrosirius red solution for 1 h at room temperature. After water wash, slides were dehydrated in ascendant concentrations of ethanol before being mounted in DPX and protect by a coverslip.

### Immunofluorescence Staining

Nunc™ Lab-Tek™8-well Chambered Coverglass (ThermoScientific) were coated with rat tail collagen and *R. pulmo* collagen. Cells were grown for 24 h and then washed twice with PBS and fixed with 4% paraformaldehyde for 15′ at RT. Cells were washed 2xPBS and permeabilized with 0.1% Triton-X 100/1x PBS for 15′ RT and then washed again 3xPBS. Blocking was performed using 3% BSA/1xPBS for 30′ RT. All the antibodies were diluted in BSA 3%. Primary antibodies (β-catenin (thermoscientific—PA5-19469) 1:100; Vinculin (Abcam—ab18058) 1:100) were incubated O.N. at 4 degree. The day after, cells were washed 3xPBS for 10′ each. Incubation with secondary antibodies was performed in dark for 1 h (antirabbit-Texas Red (life technologies—T6391) 1:400; antimouse-Texas Red (life technologies—T6390) 1:400). Cells were washed 3xPBS and finally incubated with Hoescht 33342 (Life Technologies Corporation, 1:4000/1xPBS). Image acquisition was performed on Zeiss LSM 710 confocal system.

### Statistical Analysis

All experiments had 3 biological replicates, data are shown as mean ± Standard Deviation. After normal distribution assessment data value's statistical significance was evaluated by Student's *t*-tests. Difference was considered statistically significantly at (*) p < 0.05.

## Results and Discussion

### *R. pulmo* and Mammalian Type I Fibrillar Collagen Structural Analysis

#### Electrophoretic Mobility Profile

**Figure 1:**
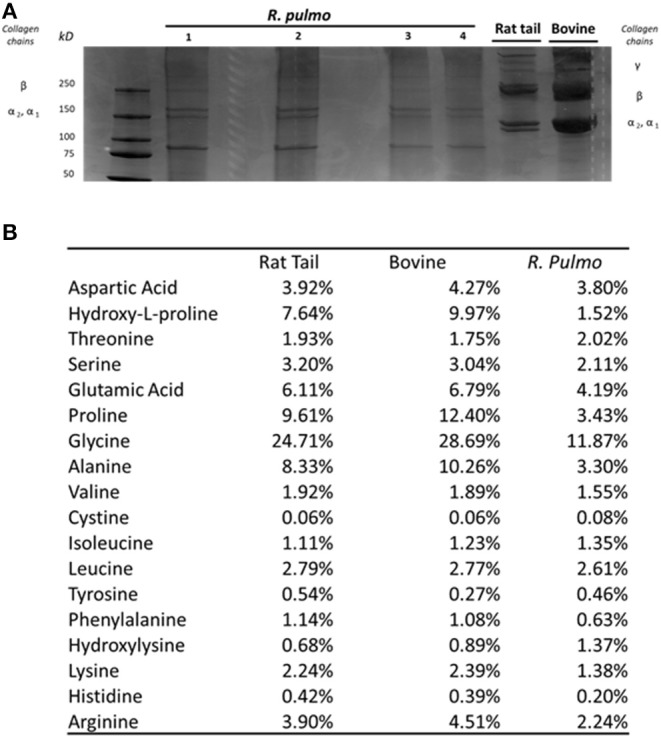
*R. pulmo* jellyfish electrophoretic banding and amino acid composition. **(A)** SDS-PAGE analysis of *R. Pulmo*, rat tail and bovine collagens*. R. Pulmo* collagen was loaded by volume: lane 1: 12 ul, lane 2: 10 ul; lane 3: 6 ul; and lane 4: 3 ul. Thirty microgram of rat tail and bovine collagen were used as controls. **(B)** Samples of acid solubilized *R. Pulmo*-rat tail-bovine collagen were compared for their amino acid composition.

SDS PAGE was used to characterize *R. pulmo* peptide chain composition. The basic structure of collagen type I is composed of three polypeptide α-chains (two α1 chains and one α2 chain), termed the coil, which are wound around each other. Under reducing conditions, *R. pulmo* collagen chain number and size were found to be present in the expected 2:1 ratio of α 1 to α 2 monomeric), with β (dimeric) and γ (trimeric) forms also being observed, and closely matched the SDS-PAGE profile of high-purity rat tail and bovine type I collagen samples, thus demonstrating very similar chain composition between type I mammalian and *R. pulmo* collagen (Figure 1A).

#### Amino Acid Composition

Collagen has a long conserved evolutionary history, and it clearly contributed to the development of early multicellular organisms (Exposito et al., 2010). Mammalian integrin collagen receptor motifs found in collagens, such as GPO (Glycine-Proline-Hydroxyproline) (Heino, 2007) and RGD (Arginine-Glycine-Aspartic acid), have been suggested as a mechanism by which mammalian cells anchor to collagens in mammals and other vertebrates (Leitinger, 2011). Collagen amino acid composition is crucial to support cell attachment and for stability and triple helix thermal behaviors. Whilst mammalian and *R. pulmo* exhibited very similar collagen chain composition, differences in amino acid content were observed. Consistent with previous reports (Song et al., 2006), *R. pulmo* collagen contained less hydroxyproline and proline, 1.52 and 3.43%, respectively, compared to rat and bovine type I collagen (Figure 1B). Whilst these amino acids are known to be important in the formation and stability of the tertiary structure (triple helix) and thermal stability properties of collagen type I, the reduced content of these amino acids did not appear to affect the structure of *R. pulmo* collagen (Sorushanova et al., 2019). Also, Glycine, Alazine, Glutamic Acid were less represented in *R. Pulmo* with 11.87, 3.30, 4.19% respectively.

#### Fourier-Transform Infrared (ATR-FTIR) Spectroscopy

**Figure 2:**
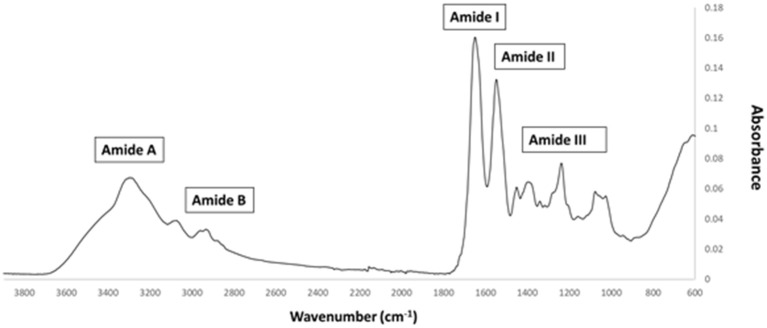
FTIR spectrum of collagen from *R. pulmo* jellyfish. Attenuated total reflection (ATR)–Fourier–transform infrared spectroscopy (FTIR) spectra reveal the collagen bands in *R. Pulmo* collagen, with collagen I specific Amide pattern.

FTIR generates a spectral fingerprint that can provide structural insights into collagen structure based on the presence and intensity of distinct peaks that correspond to amide A/B and amide I, II, and II bonds crucial to the formation of the triple helix (Belbachir et al., 2009; Riaz et al., 2018). The main absorption bands in *R. pulmo* collagen were amide A (3,283 cm^−1^), amide B (2,934 cm^−1^), amide I (1,647 cm^−1^), amide II (1,550 cm^−1^), and amide III (1,238 cm^−1^), typical bands for collagen type I (Figure 2). The amide band spectral patterns of *R. pulmo* derived collagen were comparable to mammalian collagens sources (Table 1). Amide I, II, III peak frequencies were like collagen I extracted from mammalian sources. Amide I was 1,647 cm^−1^ in *R. Pulmo*, close to 1,659 cm^−1^ of collagen from human placenta and 1,532 cm^−1^ from rat tail tendon collagen. Amide II peak was 1,550 cm^−1^, very similar to the one from human placenta 1,555 cm^−1^ and rat tail tendon 1,546 cm^−1^. Finally, Amide III showed a peak frequency of 1,238 cm^−1^ in *R. Pulmo*, compared to 1,240 cm^−1^ from human placenta and 1,243 cm^−1^ from rat tail tendon. The absorption intensity of 1,550 cm^−1^ (amide II) indicated that hydrogen bonding is present (Riaz et al., 2018); while absorption intensity of 1,238 cm-1 (amide III) confirmed that triple helical structure is intact (Riaz et al., 2018). FTIR confirmed the triple helix structure, high extent of intermolecular structure, and similar secondary structure of the proteins between different sources of collagen (Riaz et al., 2018).

**Table 1:** FTIR spectrum peaks assignment of collagen from *R. pulmo* jellyfish and comparison with mammalian type I collagen extracted from human placenta and rat tail tendon.

	**Peak frequencies (cm**^**−1**^**)**				
**Region**	***R. pulmo* jellyfish**	**Human placenta (a)**	**Rat tail tendon (b)**	**Normal range in proteins**	**Assignment**
Amide A	3,283	Ns	3,282	3,400–3,440	N-H stretch coupled with hydrogen bond
Amide B	2,934	Ns	Ns	3,100	CH_2_ asymmetrical stretch
Amide I	1,647	1,659	1,632	1,600–1,700	C = O stretch/hydrogen bond coupled with COO^**−**^
Amide II	1,550	1,555	1,546	1,510–1,580	NH bend coupled with CN Stretch, CH_2_ bend, COO^**−**^ symmetrical stretch, CH_2_ wag
Amide III	1,238	1,240	1,243	1,200–1,300	NH bend coupled with CN stretch, C-O stretch

*Ns, not shown*.

*^1^ Belbachir et al. (2009)*.

*^2^ Vidal and Mello (2011)*.

### Ovarian Cancer Cell Culture and Proliferation

The interplay between cancer cells, tissue resident cells and the surrounding extracellular matrix (ECM) strongly affect cancer tumorigenesis and progression. Specifically, the loss of integrity and homeostasis in tissue ECM is a crucial cancer hallmark, with a defined “matrisome” signature for both normal and diseased tissue demonstrating how microenvironment components are deregulated during a pathologic event (Naba et al., 2012, 2014a,b, 2017; Filipe et al., 2018; Pearce et al., 2018). 3D models aim to replicate tissue mechanical properties, providing optimal bioactive structures for cell attachment and proliferation to preserve native cellular phenotypes. Usually, matrices incorporated in current models are purified from rat, mouse and bovine sources (Felician et al., 2018). Here we investigate the biocompatibility of marine derived collagens for sponge matrix models, in terms of cellular migration, proliferation and differentiation, using OvCa cells.

#### Cell Proliferation and Morphology

**Figure 3:**
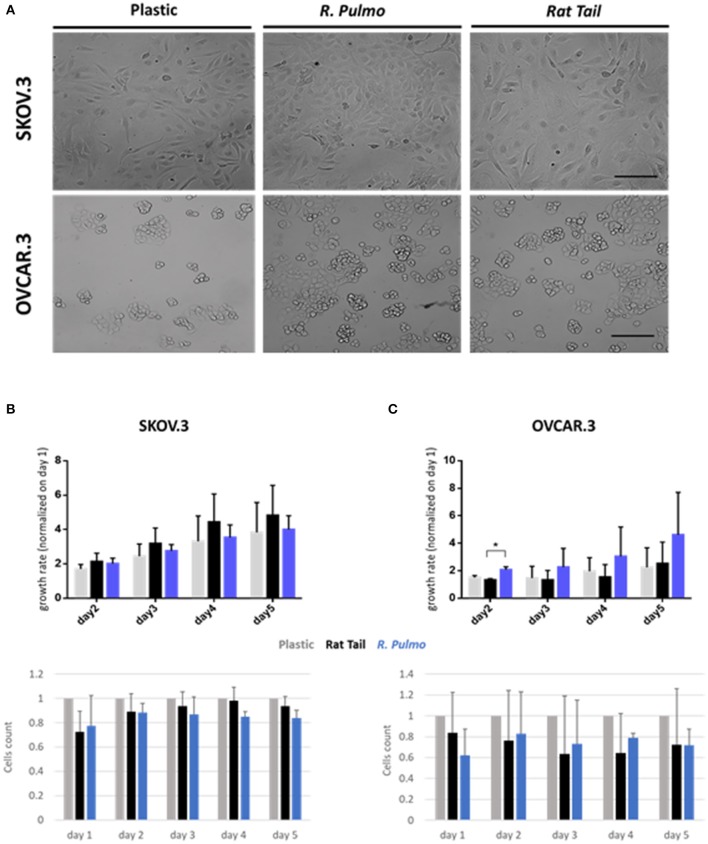
Ovarian cancer immortalized cell lines behavior on *R. pulmo* collagen substrate in 2D culture. **(A)** Brightfield acquisition after 3 days of culture, showing no cellular morphology changes after 2D OvCa cells culture on different substrates. Scale bars represent 100 um. **(B)** Cells number was obtained staining cells nuclei with Hoechst dye and analyzed with in cell analyser 2000. Analysis of growth rate after 2–3–4–5 days normalized on day 1 cells number for SKOV.3. (upper panel). Cell count comparison of cells grown on *R. Pulmo* or rat tail collagen normalized on cell number grown on plastic plates (bottom panel). **(C)** Cells number was obtained staining cells nuclei with Hoechst dye and analyzed with in cell analyser 2000. Analysis of growth rate after 2–3–4–5 days normalized on day 1 cells number for OVCAR.3. (upper panel). Cell count comparison of cells grown on *R. Pulmo* or rat tail collagen normalized on cell number grown on plastic plates (bottom panel). Data are shown as mean ± Standard Deviation (three independent experiments). ^*^Statistical significance assessed by *p* < 0.05, Student's *t*-test.

Collagen controls tissue architecture and strength while interacting with cells affecting their growth, differentiation, and migration (Ricard-Blum, 2011). During cancer progression collagen structure undergoes rearrangement becoming aligned perpendicular to the invading boundary (TACS-3), thus exerting a promigratory environment facilitating cancer migration (Adur et al., 2014; Cho et al., 2015) and is implicated in OvCa progression (Cho et al., 2015). OvCa cell lines SKOV.3 and OVCAR.3, isolated from ascites of high-grade serous carcinoma patients, had undergone total (SKOV.3) or partial (OVCAR.3) EMT (epithelial to mesenchymal transition) to colonize the ascites fluid, a vehicle for them to reach primary metastatic sites in the peritoneum (Lengyel, 2010). To evaluate biocompatibility, SKOV.3 and OVCAR.3 were cultured on *R. pulmo* collagen coated culture plates and monitored for viability (collagen cytotoxicity), proliferation and morphological changes over 5 days of culture. SKOV.3 and OVCAR3 cell morphology was not-affected by the nature of the cell culture substrate (Figure 3A), SKOV.3 retained their mesenchymal-like phenotype and OVCAR.3 showed *in vitro* epithelium morphology with a characteristic grape-like cluster pattern (Geisinger et al., 2006). Growth rate over 5 days, obtained after normalizing cell number of day 2–3 4–5 on day 1 cells' number, wasn't significantly different between cells grown on different substrates except for OVCAR.3 at day 2, which experienced a boost in proliferation on *R. Pulmo* collagen, growing 0.75 times more than rat tail substrate (*p* > 0.05) (Figures 3B,C upper panels). Compared to plastic both SKOV.3 and OVCAR.3 didn't show any intra-day difference in cell number after growing on different substrates (Figures 3B,C bottom panels).

#### OvCa Metastasis-Related Molecular Marker Expression

**Figure 4:**
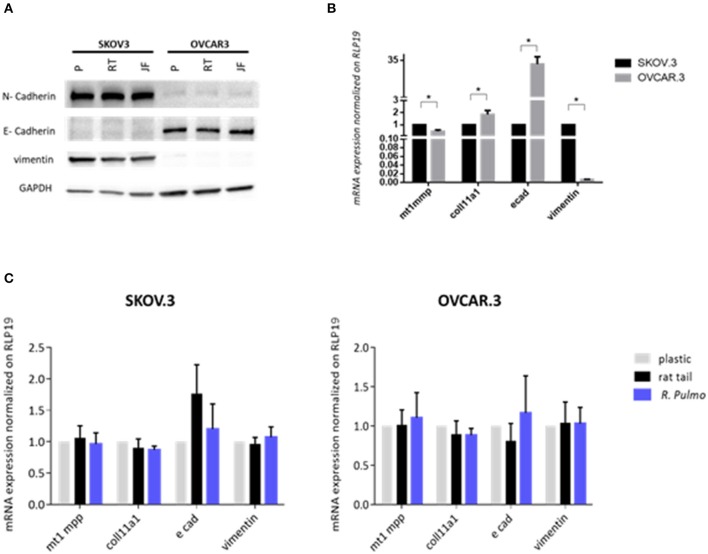
Ovarian cancer cell lines characterization. SKOV.3-highly metastatic OC cell line, is characterized by an overexpression of N-cadherin, vimentin at both **(A)** protein level and **(B)** mRNA and OVCAR.3-intermediate metastatic OC cell line, showing a typical E-cad overexpression at both **(A)** protein level and **(B)** mRNA. mRNA was extracted from cells grown on jellyfish substrate. **(C)** RT-qPCR analysis of a panel of genes related to EMT and OC progression in OvCa cells cultured on plastic, rat tail or *R. pulmo* collagen substrates. RLP19 was used as housekeeping gene and cells grown on plastic as control. AACt method was performed. Data are shown as mean ± Standard Deviation (three independent experiments). ^*^Statistical significance assessed by *p* < 0.05, Student's *t*-test.

EMT and mesenchymal to epithelial transition (MET) are cellular transformations that define metastatic cascade progression in OvCa development and differentiation (Davidson et al., 2012). To determine if *R. pulmo* collagen had any effect on EMT, the markers E-cadherin, N-cadherin and vimentin were measured at protein level and no significant difference was seen between cells grown on different substrates (*p* > 0.05) (Figure 4A). SKOV3 cells expressed high levels of N-cadherin and vimentin compared to OVCAR.3, which expressed the epithelial marker E-cadherin (Figure 4A).

The expression of pre-invasive metalloproteases is generally associated with a highly metastatic phenotype, and their expression appears fundamental for cancer cells to remodel surrounding extracellular matrix components including collagen (Krempski et al., 2012). *MT1-MMP* (MMP14), is a membrane type metalloprotease (MT-MMPs) present at high levels in OvCa cells, while other MMPs including MMP9, specific to collagens IV and V, are up-regulated in ovarian cancer stroma (Kamat et al., 2006). Furthermore, *COL11A1* expression, a component of type XI collagen, was recently associated with poor prognosis epithelial cancers including OvCa, (Wu et al., 2014). When cultured in the presence of *R. pulmo* collagen *MT1-MMP* expression was 0.53 times higher in SKOV.3 than OVCAR.3, together with vimentin, 0.99, while OVCAR.3 showed 31 times higher expression of E-cadherin and 0.84 times higher *COL11A1* compared to SKOV.3 (*p* > 0.05) (Figure 4B). Comparing EMT markers mRNA expression levels of cells cultured on *R. Pulmo* and rat tail collagen to plastic substrate we didn't find any significant difference (*p* > 0.05) (Figure 4C). Unaltered mRNA expression of those markers further confirm that marine collagen can effectively substitute for mammalian collagen in 2D *in vitro* cell studies.

#### *R. pulmo* Collagen Substrate Cell Adhesion

**Figure 5:**
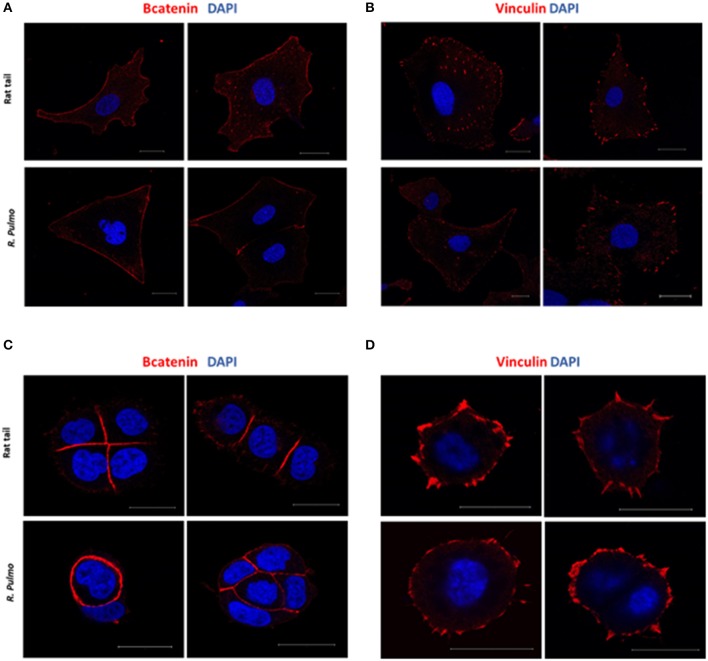
Adherents junctions and focal adhesion assembly in OvCa cells grown on *R. pulmo* and rat tail collagen substrate. SKOV.3 immunostaining of **(A)** B-catenin and **(B)** vinculin cells plated onto rat tail type I collagen, and *R. pulmo* jellyfish collagen. OVCAR.3 immunostaining of **(C)** B-catenin and **(D)** vinculin cells plated onto rat tail type I collagen, and *R. pulmo* jellyfish collagen. Scale bars represent 20 um.

In a normal epithelium both cell-cell interaction and connections with the underlying basement membrane govern tissue structure (Yurchenco, 2011). Cell junctions contain a number of multiprotein complexes that connect neighboring cells (Cooper, 2000) including Adherent Junctions (AJ), which contain cadherins that anchor intracellular actin filaments with intercellular of adjacent cell bridged by β-catenin (Takeichi, 1991). In the presence of either rat tail or *R. pulmo* collagen β-catenin was distributed uniformly across the cell membrane in SKOV.3 cells (Figure 5A), whereas in OVCAR.3 β-catenin predominately localized at cell-cell junctions (Figure 5C). Similarly, vinculin (Addad, 2011), a component of focal adhesion complexes linking cells to basement membranes, was expressed in the membranes of cells grown on both collagen types (Figures 5B–D).

#### *R. pulmo* Collagen Sustains and Supports OvCa 3D Cell Culture

Among 3D *in vitro* models, monocellular spheroids and scaffold-based 3D tumor cultures are the simplest and most widely used (Ricci et al., 2013). Spheroids replicate cell-cell interaction in the tumor, show increased chemoresistance, gradients of diffusion, hypoxic core as well as partial secretions of ECM (Burdett et al., 2010). More significant addition of cell-matrix interactions is achieved using scaffold-based 3D culture such as acellular 3D matrix or a liquid hydrogel matrix mixed with cells followed by solidification or polymerization. Some hydrogel specific limitations exist, such as insufficient porosity for long term cell culture and the promotion of accurate ECM deposition, furthermore many hydrogel constituents are synthetic with limited functionality and utility for ECM–cell communication (Horvath et al., 2016).

#### Cell Migration Through *R. pulmo* Collagen Scaffolds

**Figure 6:**
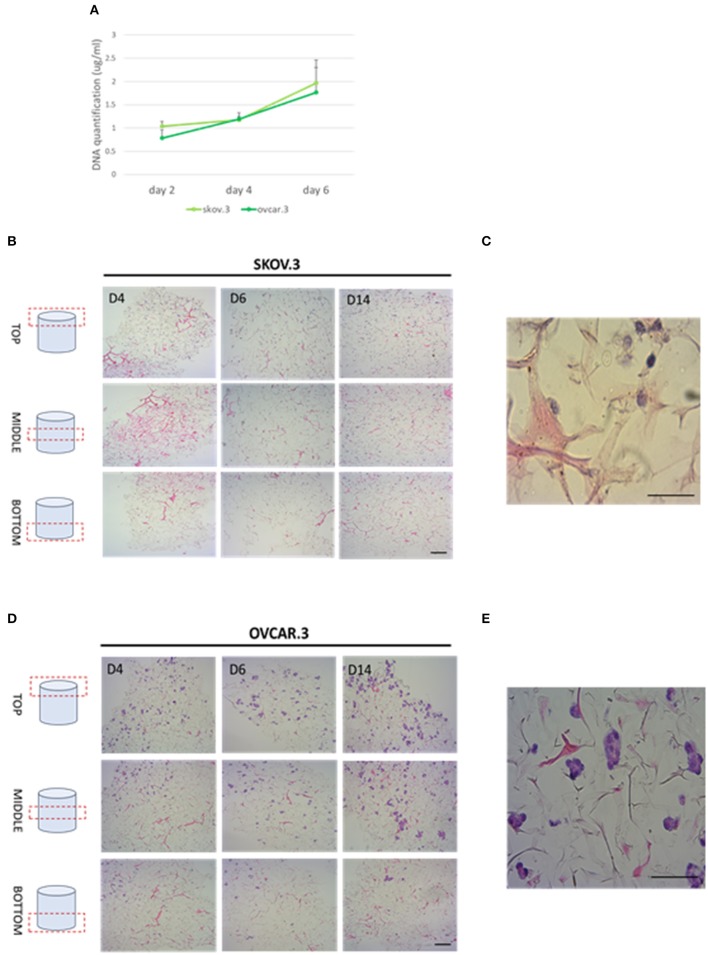
Cell proliferation and migration through a 3D-jellyfish collagen scaffold. **(A)** SKOV.3 and OVCAR.3 cells proliferation rate on 3D jellyfish scaffold from day 2 to 6 assessed through DNA quantification using PicoGreen® dsDNA quantitation assay. **(B)** SKOV.3 cells were seeded on the top of the scaffold and they migrated and colonized the entire scaffold to the bottom from day 4 to 14. **(C)** High magnification of SKOV.3 cells directly interacting with collagen as single cells. **(D)** OVCAR.3 cells were seeded on the top of the scaffold and they migrated and colonized the entire scaffold to the bottom from day 4 to 14. **(E)** High magnification of OVCAR.3 cells directly interacting with collagen as cluster of cells. Scale bars indicate 100 um.

Collagen-based sponge scaffold systems, shaped in different ways, are widely used in biomedicine. Those devices reflect basic features of tissue structure and organization, trying to recapitulate closer tissue complexity. Hydrogels, sponges, fibers, and films have been developed as biocompatible tissue engineered substitutes for tissue grafts, reparative medicine (Sorushanova et al., 2019). Freeze drying is a common method used to obtain highly porous implantable sponge devices for use in clinical applications including bone repair and wound healing (Sorushanova et al., 2019). Freezing rates can be controlled to manipulate sponge pore size, where high pore size can enhance cell migration and nutrient diffusion, whilst smaller pore sizes increase cell adhesion (Sorushanova et al., 2019). Having confirmed the biocompatibility of *R. pulmo* collagen, we investigated its utility as material for producing sponge scaffolds. Sponge scaffolds were produced using a freeze-dried protocol (Hoyer et al., 2014) and molded into a cylindrical shape using a 96 well plate, reporting a final diameter of 5 mm (Supplementary Figure 1A). SEM analysis of scaffold porosity showed that fabricated sponges had an average porosity of 98 nm +/−11.33 (Supplementary Figure 1B) with an ordinated pore structure (Supplementary Figure 1C). Picro Sirius red staining stained specifically *R. Pulmo* collagen unveiling its 3D collagen fibers' arrangement (Supplementary Figure 2). *R. pulmo* sponge scaffolds were seeded from the top surface with SKOV.3 and OVCAR.3 at a density of 2 × 10^5^ cells per scaffold, and cells' proliferation across collagen scaffold was examined at day 2–4–6. Both cell lines grew on *R. Pulmo* collagen scaffold showing doubled DNA amount at day 6 compared to day 2, indicative of cells proliferation (Figure 6A). Cells distribution across *R. pulmo* collagen scaffold was analyzed at day 2–4–6. Notably, at day 14 both SKOV.3 and OVCAR.3 cells were found to have successfully invaded and colonized the entire scaffold, from the top to the bottom section (Figures 6B–D). During migration and proliferation SKOV.3 grew as single cells, while OVCAR.3 formed cell clusters (Figures 6C–E). We concluded that this collagen supports the development of a bioactive network enabling OvCa cell migration.

#### OvCa Metastasis-Related Markers Expression in 3D Scaffolds Compared to 2D

**Figure 7:**
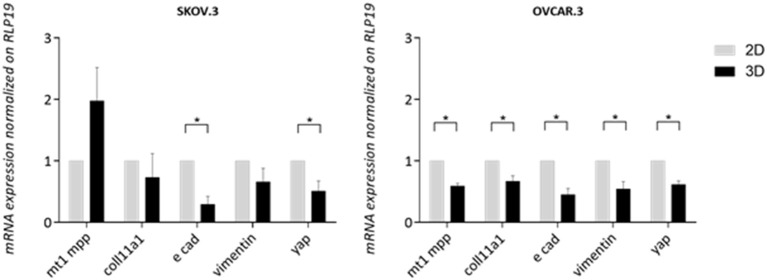
Transcript level expression of EMT and OvCa progression markers in 3D scaffolds compared to 2D systems. RT-qPCR analysis of a panel of genes related to EMT and OC progression. RLP19 was used as housekeeping gene and cells grown on 2D system as control. AACt method was performed. Data are shown as mean ± Standard Deviation (3 independent experiments). Statistical significance assessed by ^*^p < 0.05, Student's *t*-test.

A panel of metastasis/EMT-related markers were evaluated to understand if the scaffold promoted or repressed cancer cell metastatic properties. Gene expression of EMT-related markers was highly influenced by a 3D environment with a widespread lower expression of most genes compared to a simple 2D system. E-cadherin showed a difference of 0.70 lower expression in SKOV.3 cells grown on 3D scaffold compared to 2D scaffold, suggesting a possible strengthening of the metastatic phenotype. OVCAR.3 wide lower expression of *MT1MMP, COLL11A1*, vimentin,0.40, 0.33, 0.45, respectively, on 3D compared to 2D culture seems to suggest the acquisition of an even less metastatic phenotype with the only exception of E-cad which was also downregulated 0.45 times in 3D compared to 2D (*p* > 0.05) (Figure 7). Finally, YAP1, a transcriptional factor and mechano-transducer involved in the initiation, progression, and metastasis of several cancers (Zanconato et al., 2016; Quintela et al., 2019), was downregulated 0.49 times in SKOV.3 and 0.55 in OVCAR.3 cells cultured on 3D scaffolds compared to 2D culture, suggesting that multidimensional culturing methods strongly influence ovarian cancer gene expression compared to 2D systems (*p* > 0.05) (Figure 7).

Low success rates in OvCa specific drug discovery are linked, in part, to the lack of advanced preclinical *in vitro* screening methods (Ocana et al., 2010; Hutchinson and Kirk, 2011). Many mouse models have been used to interrogate the complexities of ovarian cancer, with orthotopic and humanized mouse models aiding the understanding of ovarian tumorigenesis and immunotherapy (Fong and Kakar, 2009; Bobbs et al., 2015; Hasan et al., 2015). Animal models can mimic some, but not all, of the important facets of human responses. Indeed, only 5% of potential anticancer drugs tested in mice reported sufficient clinical activity in phase III clinical trials to eventually be licensed.

3D *in vitro* models such as those shown here, can help overcome the high cost and the time-consuming nature of *in vivo* studies. Existing 3D models for breast cancer and melanoma (Berking and Herlyn, 2001; Weigelt et al., 2014) have provided a foundation for the design of OvCa 3D culture models (Watters et al., 2018). High-grade serous OvCa tumorigenesis models comprises *ex vivo* fallopian tube models, *in vitro* fallopian tube spheroid models, organ cultures using alginate hydrogels, with disease progression and metastasis often studied on 3D organotypic culture models that aim to recapitulate the OvCa omental and peritoneal tumor microenvironment (White et al., 2014).

More complex co-culture (e.g., with cancer cells, stromal cells, macrophages) 3D model systems such as multicellular tumor spheroids alone or imbedded in hydrogels systems or on dried scaffolds are now becoming available (Brooks et al., 2019). The hydrogel source can be naturally derived (collagen, fibrin, hyaluronic acid, matrigel and derivatives of natural materials) or synthetic, including polyvinyl alcohol (PVA), polylactide-co-glycolide (PLG), polycaprolactone (PLA) and polyethylene glycol (PEG) hydrogels. The second category offers more flexibility in tuning chemical and mechanical properties (Horvath et al., 2016). Moreover, those materials can be functionalized with specific peptides to explore ECM turnover and interaction with tumor cells (Peyton et al., 2018). The inclusion of fibroblasts, immunity cells and vasculature in a 3D tumor-stroma system will help elucidate metastasis mechanisms, chemoresistance and the interaction between mechanical properties, topology, and matrix composition to promote cancer survival and dissemination (Valkenburg et al., 2018).

## Conclusion

The major challenge of this century is understanding cancer biology. Reproducing tumor complexity in an *in vitro* model doesn't exclusively require malignant cells but it must replicate the microenvironment which can constrict or nurture the tumor mass.

Nowadays, many investigations are still performed on cell monolayers, excluding the environment effect on cancer development. Although those 2D models are highly reproducible they lack in tumor self-protecting mechanism involving cell-cell and cell-matrix interactions, they don't mimic drug penetration, ECM is absent and cell phenotype is very different from their *in vivo* counterpart. Consequently, 2D platforms provide misleading information on drug delivery, efficiency and selectivity and don't replicate accurately cancer complexity. A truthful model should provide a tool with tuneable properties which can more closely reproduce the tumor microenvironment. In the last 10 years, starting in 2006, the necessity for complex biomimetic models has led to the design of new 3D cancer models where complex cell-cell and cell-extracellular matrix (ECM) interactions can develop in a biomimetic fashion (Ingber et al., 2006).

Marine organisms represent an attractive new source of collagen not least because they could address the global imperative for developments that lead to the reduction in the use of animals and their derived products in research (Balls, 2009). Adopting jellyfish as a collagen source is sustainable and cheaper than the golden standard material sources derived from mammals (rat tail and bovine). Furthermore, jellyfish derived collagen is tuneable, with specific functionalization possible, to perform investigations on cancer/stroma interactions that occur in both tumorigenesis and metastasis. In this study we demonstrated that *R. pulmo* collagen represents an excellent substrate for use in 2D and 3D *in vitro* cell culture. Structural and biological analysis demonstrated that *R. pulmo* derived scaffolds performed comparably with rat tail and bovine-derived collagen type I. Electrophoretic mobility in SDS-PAGE showed only minor differences in α-helixes band patterning with a high degree of conservation of triple helix organization. Whilst hydroxyproline, proline, glycine, and arginine content was lower than that reported for rat tail and bovine collagens (Song et al., 2006), the ability to support cell growth and interaction as well as basement substrate adherence suggests *R. pulmo* collagen is functionally analogous, and that it is likely to contain GPO and RGD signatures. Alternatively, whilst collagens are found in all metazoans and are considered to have contributed to the early evolution of multicellular animals (Exposito et al., 2010), collagen receptors appeared much later, therefore *R. pulmo* collagen could provide different cell adhesion sites compared to mammalian sources.

Using metastatic OvCa cell lines established from ascites samples that are capable of collagen type I adherence, migration and remodeling, both SKOV.3 and OVCAR.3 cells were able to proliferate normally on *R. pulmo* (and rat tail) collagen coated plates. No cytotoxicity was noted and cell morphology and metastatic potential, evaluated through a panel of EMT-related markers, was not altered by the underlying substrate. Similarly, identical focal adhesions assembled on all substrates tested.

3D models offer the potential to mimic the dense matrix network associated with tumor microenvironments, providing a physiologically relevant tool for biomedical research and preclinical drug testing. Molded *R. pulmo* sponges provided excellent support for cancer cell growth in a type I-like collagen environment that is known to be a major ECM component in both normal and cancer tissues. OvCa cells exhibited different invasion/growing-pattern through *R. pulmo* collagen, as single (highly metastatic SKOV3) or as clusters cells (low metastatic OVCAR3). Both cell types colonized the full extent of the collagen network and displayed altered expression of some EMT-related markers in a 3D environment compared to 2D culture. *R. pulmo* provides an alternative collagen source that can be produced at scale, and which replicates the functionality of mammalian collagen in both 2D and 3D *in vitro* systems.

## Data Availability Statement

All datasets generated for this study are included in the article/Supplementary Material.

## Author Contributions

FP carried out all the experiments and wrote the manuscript. JF carried out the aminoacid sequencing experiment. SY supervised the design of few experiments. FB helped supervise the project. FT worked on the manuscript. DG supervised the project. RC supervised the project and revised the manuscript. LF conceived the original idea and aided in interpreting the results.

### Conflict of Interest

The authors declare that the research was conducted in the absence of any commercial or financial relationships that could be construed as a potential conflict of interest.
