# Supplementary material for: Marine Collagen Substrates for 2D and 3D Ovarian Cancer Cell Systems
Source: Front Bioeng Biotechnol. 2019 Dec 13;7:343. doi: 10.3389/fbioe.2019.00343 (PMC6923181; doi:10.3389/fbioe.2019.00343)
Supplement: Supplementary file 1 [file Table_1.DOCX]

**SUPPLEMENTARY DATA**

**Supplementary Figure 1.**

**
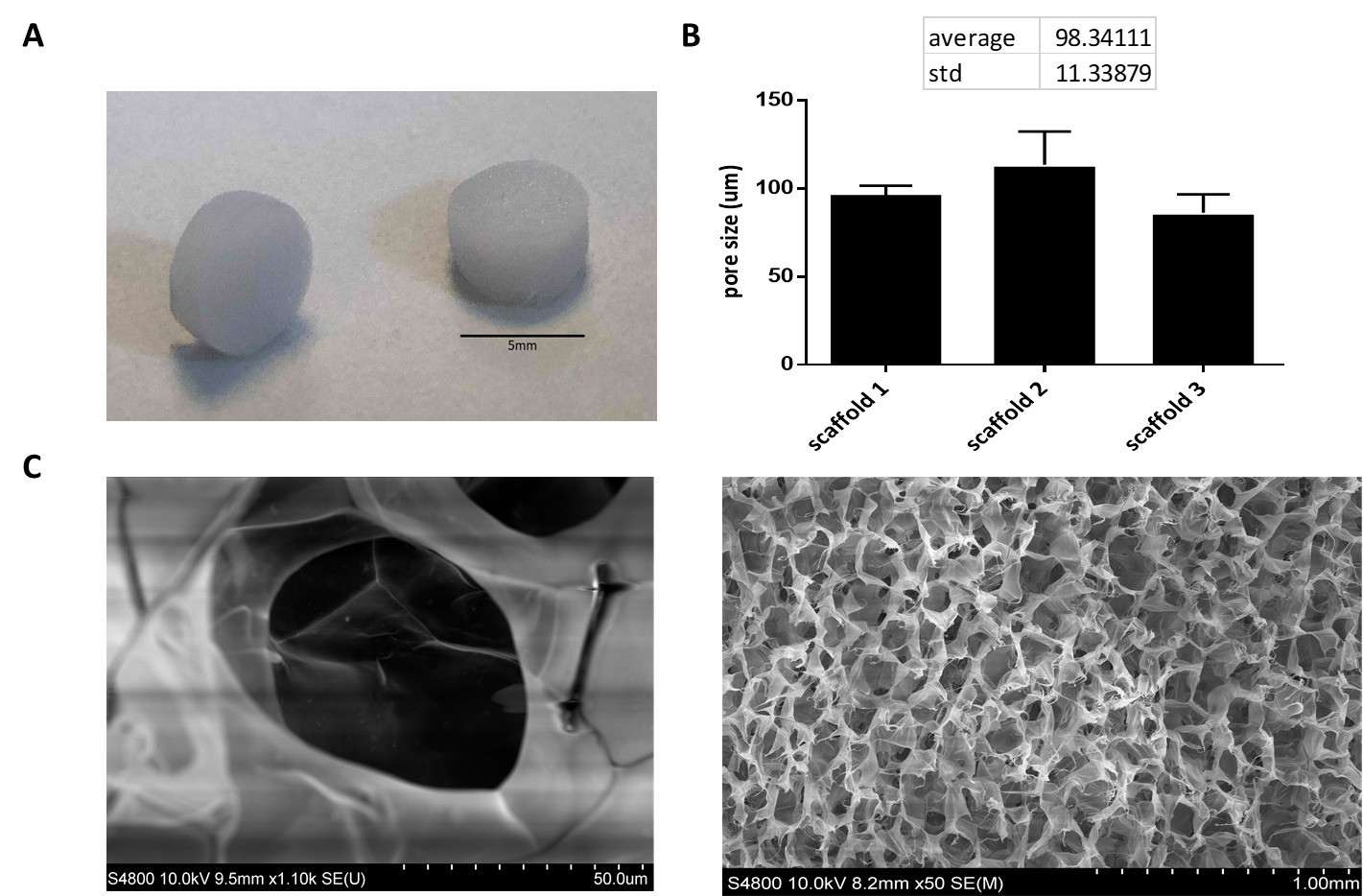
**

**Supplementary figure 1. 3D R. Pulmo collagen scaffold pore characterization.** (A) Picture of scaffold sponge moulded on 96 well plate (diameter of 5 mm) (B) SEM analysis of scaffold porosity. (C) SEM analysis of scaffold pore structure and pores arrangement. *Data shown as mean ± Standard Deviation (3 independent scaffold).*

**Supplementary Figure 2.**

**
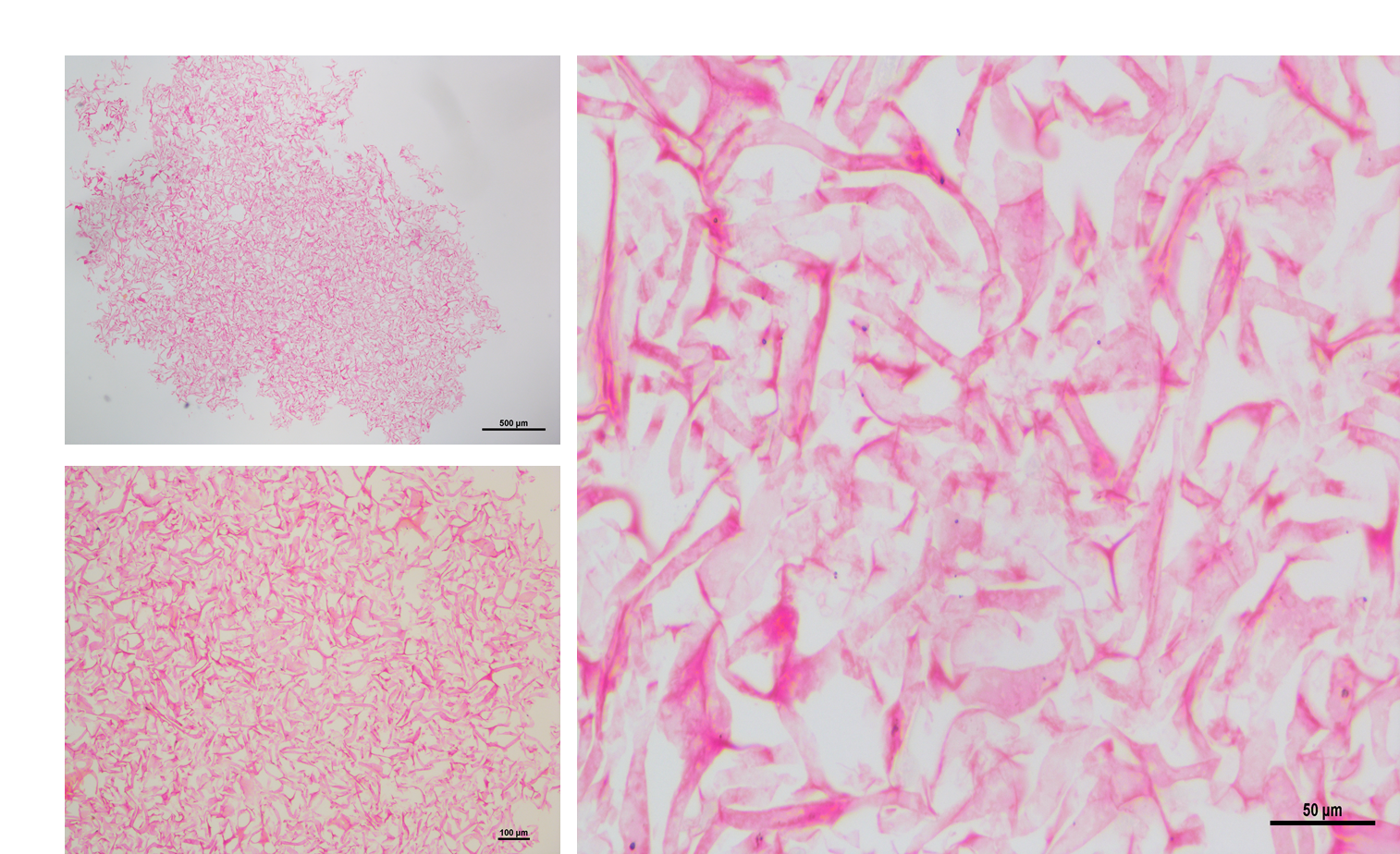
**

**Supplementary figure 2. 3D R. Pulmo collagen scaffold structure.** Picro Sirius red staining of a jellyfish collagen scaffold showing collagen fibres arrangement at different magnifications.
